# Supplementary material for: Primary Production in the Kara, Laptev, and East Siberian Seas
Source: Microorganisms. 2023 Jul 26;11(8):1886. doi: 10.3390/microorganisms11081886 (PMC10456892; doi:10.3390/microorganisms11081886)
Supplement: Supplementary file 1 [file microorganisms-11-01886-s001.zip › microorganisms-2392000-supplementary.pdf]

Table S1. Total chl-*a* concentrations, hourly primary production rate, and freshwater content of each sampling station in the Kara Sea, the Laptev Sea, and the East Siberian Sea.

Region	Year	Station	Total chl-*a* (mg chl-*a* m^{-2})	Primary production rate (mg C $m^{-2}h^{-1}$)	Freshwater content (m)
**Kara Sea**	**2013**	AF091	57.5	9.2	2.0
		AF095	20.4	3.6	3.5
		AF100	29.2	4.9	2.6
		AF116	11.7	1.0	1.5
	**2015**	AT003	30.9	3.6	1.7
		AT079	9.9	2.3	2.2
		AT088	10.3	4.4	3.7
**Laptev Sea**	**2013**	AF005	23.1	0.4	2.7
		AF006	26.9	5.9	3.2
		AF011	26.1	4.6	4.8
		AF019	44.1	17.6	3.0
		AF024	11.3	1.6	4.6
		AF036	9.9	3.9	6.9
		AF049	58.0	4.0	5.2
		AF057	25.1	4.1	4.3
		AF061	39.2	6.1	3.1
		AF068	24.3	4.8	2.0
		AF071	13.4	1.2	4.2
		AF072	15.5	3.0	3.0
		AF080	15.3	2.0	1.9
	**2015**	AT014	20.5	4.5	3.7
		AT022	17.3	4.8	4.1
		AT029	17.8	4.4	3.7
		AT034	14.7	2.4	0.6
		AT074	11.7	1.9	3.9
		AT077	13.6	3.6	2.5
	**2018**	AT18-28	23.9	0.3	3.2
		AT18-31	16.7	2.7	3.7
		AT18-35	26.9	7.4	2.6
		AT18-42	12.1	6.4	2.8
		AT18-47	7.8	4.6	6.2
		AT18-55	16.2	1.6	3.8
		AT18-59	13.1	4.6	5.9
		AT18-108	13.6	5.6	5.2
		AT18-122	20.6	3.0	2.6
**East Siberian Sea**	**2013**	AF041	13.5	2.5	7.5
		AF044	9.9	3.0	7.6
	**2015**	AT041	15.2	2.4	5.6
		AT046	20.6	5.8	11.4
		AT053	16.6	2.4	11.5
		AT060	14.1	2.1	15.8
		AT066	19.7	1.5	17.1
		AT071	18.7	5.2	11.0
	**2018**	AT18-66	6.9	1.4	7.5
		AT18-70	5.2	0.8	4.8
		AT18-75	6.0	0.7	9.8
		AT18-81	10.7	2.0	9.5
		AT18-85	7.8	2.7	10.1
		AT18-96	7.6	1.5	7.6
		AT18-97	9.5	1.1	7.9
